# HtrA4 may play a major role in inhibiting endothelial repair in pregnancy complication preeclampsia

**DOI:** 10.1038/s41598-019-39565-9

**Published:** 2019-02-25

**Authors:** Yao Wang, Rebecca Lim, Guiying Nie

**Affiliations:** 1 Implantation and Placental Development Laboratory, Centre for Reproductive Health, Hudson Institute of Medical Research, Clayton, Victoria 3168 Australia; 2 Department of Molecular and Translational Science, Monash University, Clayton, Victoria 3800 Australia; 3 Department of Obstetrics and Gynaecology, Monash University, Clayton, Victoria 3168 Australia; 4 Australian Regenerative Medicine Institute, Monash University, Clayton, Victoria 3168 Australia; 5 Department of Biochemistry and Molecular Biology, Monash University, Clayton, Victoria 3800 Australia

## Abstract

Preeclampsia (PE) is a life-threatening complication of human pregnancy with no effective treatment other than premature delivery. It is hallmarked by systemic endothelial injury/dysfunction which is believed to be caused by abnormal levels/types of placenta-derived factors that are circulating in the maternal blood. Emerging evidence suggests that endothelial repair is also dysregulated in PE, as circulating endothelial progenitor cells (EPCs) critical for endothelial regeneration are reduced in number and functionality. However, the underlying mechanisms are poorly understood. HtrA4 is a placenta-specific protease that is secreted into the circulation and significantly elevated in early-onset PE. Here we investigated the impact of HtrA4 on endothelial proliferation and repair. We demonstrated that high levels of HtrA4 halted endothelial cell proliferation and significantly down-regulated a number of genes that are critical for cell cycle progression, including *CDKN3*, *BIRC5*, *CDK1* and *MKI67*. Furthermore, HtrA4 significantly inhibited the proliferation of primary EPCs isolated from term human umbilical cord blood and impeded their differentiation into mature endothelial cells. Our data thus suggests that elevated levels of HtrA4 in the early-onset PE circulation may impair endothelial cell repair, not only by halting endothelial cell proliferation, but also by inhibiting the proliferation and differentiation of circulating EPCs.

## Introduction

Preeclampsia (PE) is a serious disorder of human pregnancy that affects 2–8% of pregnancies worldwide^1,2^. PE is characterized by a *de novo* hypertension accompanied by proteinuria and/or organ dysfunction^3,4^. The condition can progress rapidly leading to multi-organ failure, with symptoms closely linked to wide-spread endothelial dysfunction^5^. Currently, the only effective treatment of PE is the premature delivery of the fetus, along with the problematic organ – the placenta.

PE can be classified into two distinct subtypes: early-onset which occurs before 34 weeks of gestation, and late-onset which occurs after 34 weeks^6^. The two PE subtypes may have different aetiologies. Early-onset PE is associated primarily with inadequate trophoblast invasion during early placentation, which leads to placental ischemia and reduced blood supply to the foetus later in pregnancy^7,8^. Late-onset PE is less likely linked to abnormal trophoblast invasion, suggesting that other factors are involved in the disease development^9^.

Early-onset PE poses far more significant maternal risks, with significant higher mortality rate compared to late-onset PE^10,11^. The risk of cardiovascular disease is also much higher in women who have had early-onset than late-onset PE^12–14^, suggesting that endothelial dysfunction is more profound in early-onset PE and persists long after the pregnancy^15^. Markers of endothelial dysfunction such as vascular cell adhesion molecule (VCAM)-1 and intercellular adhesion molecule (ICAM)-1 remain elevated in women even 15 years after their preeclamptic pregnancy^16^. This is consistent with the view that endothelial dysfunction resulting from PE may account for the increased risk of cardiovascular diseases in women with a history of preeclamptic pregnancies^17^. These data suggest that early-onset PE has a long-lasting effect on endothelial cells that is not restored after the symptoms of PE have been resolved.

Endothelial progenitor cells (EPCs) are a unique population of cells that circulate in the blood, and are recruited to the endothelium upon endothelial injury, where they then differentiate into resident endothelial cells to regenerate the blood vessels and restore endothelial function^18,19^. In the non-pregnant population, reduction in circulating EPCs is associated with increased cardiovascular risks, highlighting the importance of EPCs in the maintenance of endothelial function^20^. EPC numbers and migratory activities are inversely correlated to risk factors of coronary artery disease^21^. Notably, EPCs isolated from patients with type II diabetes have impaired proliferation, adhesion and angiogenic activity^22^. In normal human pregnancy, the maternal endothelium undergoes extensive remodelling and repair, where circulating EPCs are suggested to play a major role in endothelial repair^23–25^. One study has reported that EPC numbers progressively increase in normal pregnancy and the highest levels are detected in the third trimester^26^. The same study has also demonstrated that circulating EPC numbers in the third trimester are significantly lower in pregnancies that are complicated by intrauterine growth restriction^26^. Other studies have reported that maternal as well as fetal/placental EPCs are significantly reduced in PE^27–29^. EPCs isolated from umbilical cord blood of preeclamptic pregnancies have impaired proliferation, migration and vasculogenesis in culture^27^. Furthermore, circulating EPCs in early-onset PE are reported to exhibit increased senescence^30^. These studies suggest that EPCs may play an important role in normal pregnancy but they are reduced in number and functionality in PE. However, it is unknown how EPCs are compromised in PE.

It is well established that in PE the placenta releases abnormal types/amounts of factors into the maternal circulation, which contribute to endothelial dysfunction and the maternal syndrome of PE^31^. Factors that are significantly elevated in the PE circulation include cytokines, antiangiogenic factors, syncytiotrophoblast microparticles and activated leukocytes^32–35^. Some of these are shown to induce endothelial injury and dysfunction, especially in the case of early-onset PE^31^. However, whether these circulating placental factors compromise EPCs in PE is not well understood.

We have previously reported that high temperature requirement factor A4 (HtrA4) is a placenta-specific serine protease that is released into circulation and significantly increased in early-onset PE^36^. HtrA4 belongs to a serine protease family that serves as ATP-independent protein quality control factors in regulating cell growth, unfolded stress response, and aging^37^. HtrA4 contains a trypsin-like serine protease domain, and a highly conserved C-terminal PDZ domain which regulates protein-protein interaction^38^. In a normal human pregnancy, serum HtrA4 level increases progressively to around 24–25 weeks of gestation, then remains relatively stable throughout the remainder of the pregnancy^36^. However, the exact role of HtrA4 in placental development remains unclear. To date, two studies suggest that HtrA4 may directly regulate trophoblast function, but the results are somewhat conflicting as one shows that HtrA4 promotes trophoblast invasion^39^, whereas the other reports that HtrA4 inhibits trophoblast cell migration and growth^40^. In early-onset PE, placental expression as well as circulating levels of HtrA4 are significantly increased at the time of disease presentation^36,40–44^. We have further demonstrated that elevated concentrations of HtrA4 detected in early-onset PE circulation disrupt human umbilical vein endothelial cell (HUVEC) tube formation and induce pro-inflammation^36,45^. These results suggest that high levels of circulating HtrA4 of placental origin may dysregulate maternal endothelial cell function and contribute to the development of early-onset PE.

The aim of this study was to investigate the impact of elevated HtrA4 in the circulation of early-onset PE on maternal endothelial cell proliferation and repair. We first used HUVECs as an endothelial model, then validated the data in primary EPCs. Since EPCs from maternal and fetal origin exhibit similar phenotypes and characteristics once they are in culture^46^, we isolated EPCs from umbilical cord blood of term human pregnancy and used as an EPC model for the current study.

## Results

### HtrA4 inhibits HUVEC proliferation

**Figure 1:**
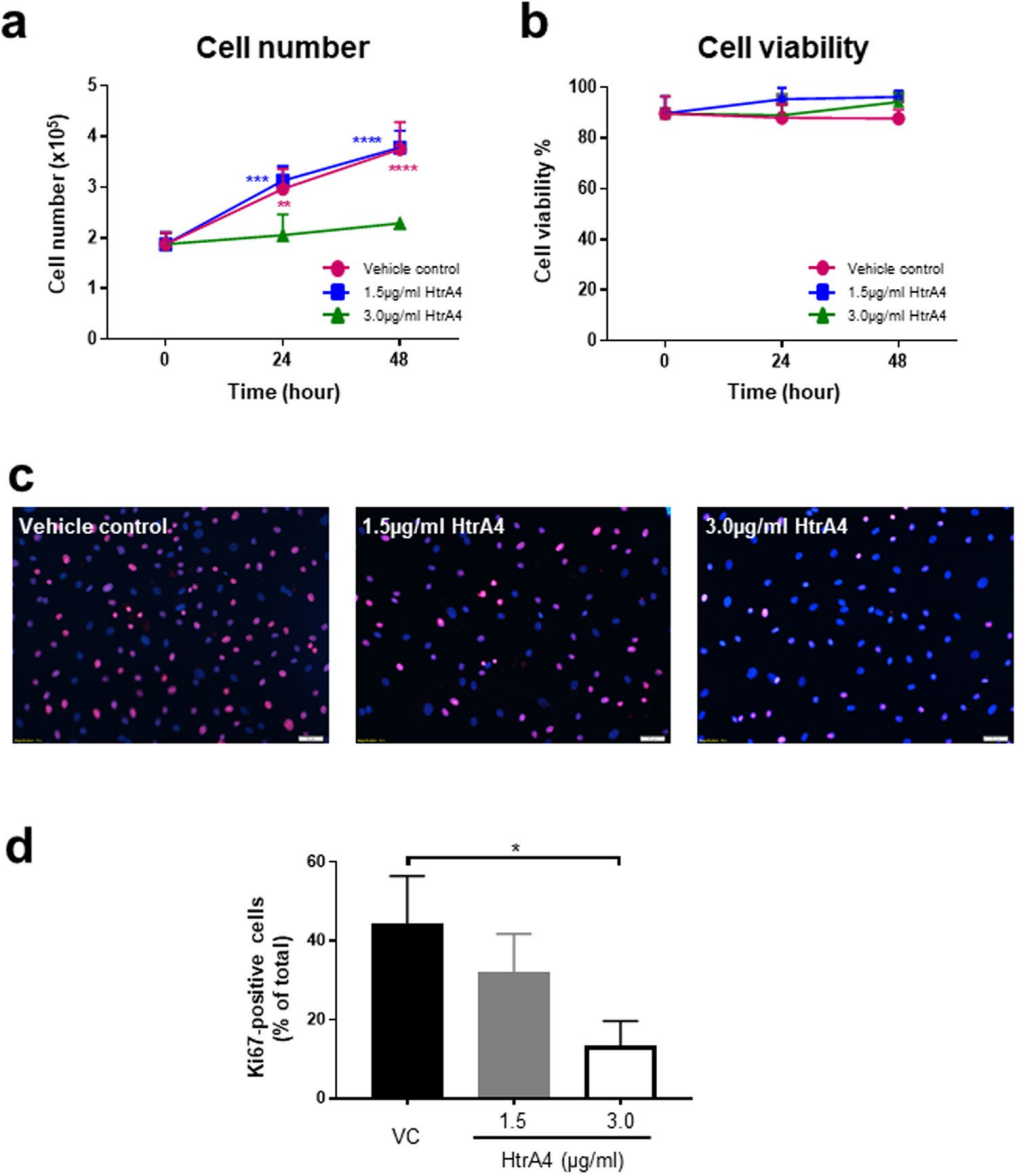
HtrA4 inhibits HUVEC proliferation. HUVECs were treated with vehicle control or two doses of HtrA4 over 48 h. (**a**) Growth curve. (**b**) Percentage of viable cells. (**c**) Immunofluorescence of cell proliferation marker Ki67 at 24 h, representative images at 10x magnification are shown. (**d**) Quantification of Ki67 staining of (**c**). n = 3. Data is expressed as mean ± SD, ^*^p < 0.05, ^**^p < 0.01, ^***^p < 0.001, ^****^p < 0.0001.

To assess proliferation, HUVECs were treated with vehicle control or two doses (3.0 ug/ml and 1.5 ug/ml) of HtrA4 for 0, 24 and 48 h, and cell number was counted at each time point (Fig. 1a). The two HtrA4 doses were chosen to represent the median and highest levels of HtrA4 detected in the early-onset PE circulation^36^. Cells treated with vehicle control steadily increased number over the 48 h period as expected (Fig. 1a). While 1.5 µg/ml HtrA4 had no obvious effect on cell growth, 3.0 µg/ml HtrA4 significantly inhibited cell proliferation (Fig. 1a). Cells from all three treatment groups were equally viable (Fig. 1b), suggesting that HtrA4 did not induce cell death while inhibiting proliferation.

We next examined the expression of cell proliferation marker Ki67 by immunocytochemistry (Fig. 1c). The majority of the cells in the control group showed clear positive nuclear staining of Ki67. However, the intensity of Ki67 staining was much lower in HtrA4-treated cells (Fig. 1c). Quantification showed that HtrA4 dose-dependently reduced Ki67 staining, and that 3.0 µg/ml HtrA4 significantly reduced the number of Ki67-positive cells (Fig. 1d).

### HtrA4 suppresses cell cycle gene expression in HUVECs

**Figure 2:**
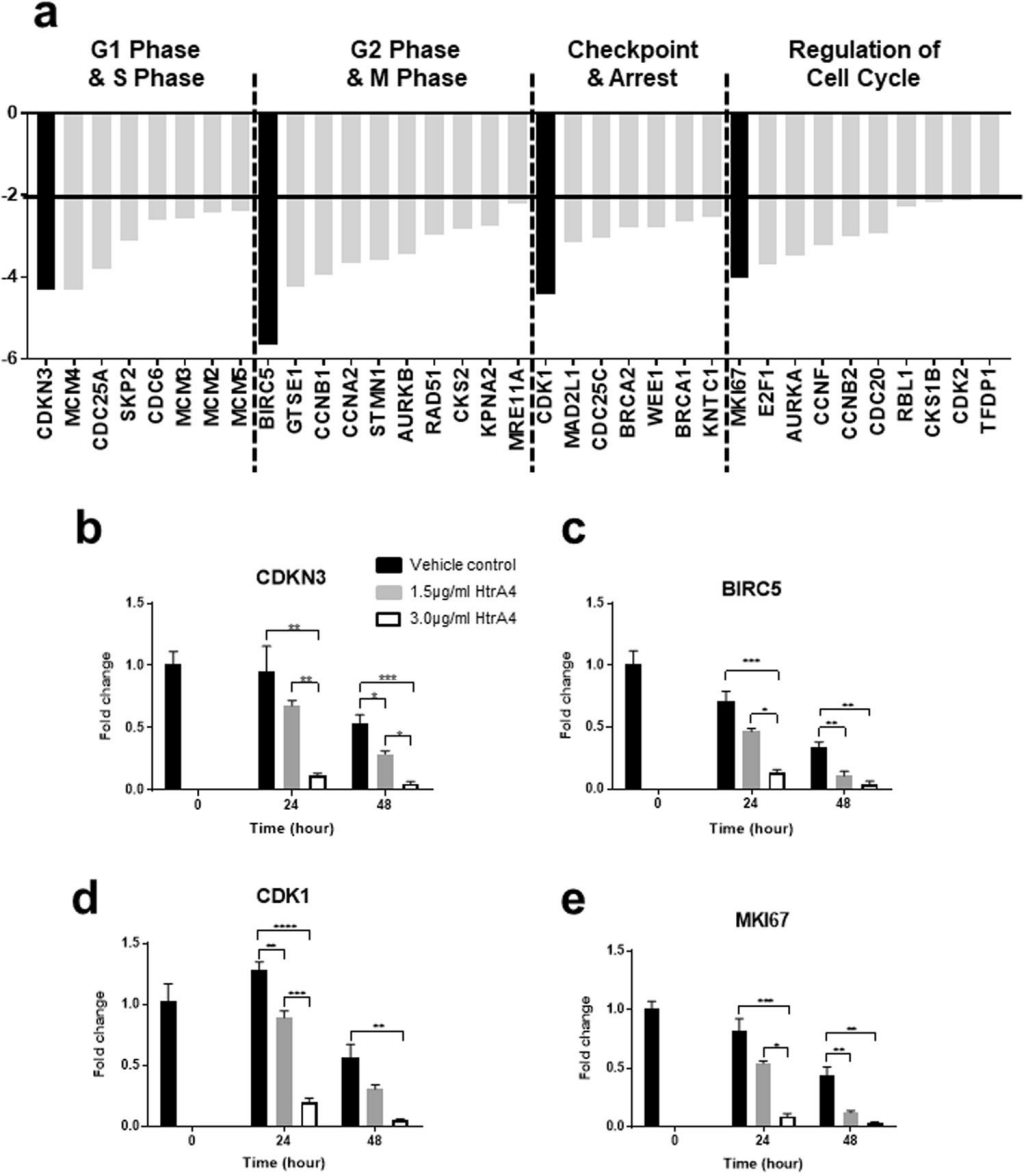
HtrA4 suppresses HUVEC mRNA expression of cell cycle genes. (**a**) Summary of PCR array analysis of 84 cell cycle genes. RNA from cells treated with vehicle control or 3.0 μg/ml HtrA4 for 24 h were pooled from 3 independent experiments and applied to the array. Data is expressed as HtrA4-induced fold changes relative to the vehicle control, and 35 genes that displayed a greater than 2-fold reduction in expression are categorised into four groups. These 35 genes were further validated by real-time RT-PCR, data of those represented in black bars are shown in (**b-e**) and the remainder are shown in Supplementary Fig. 1A–D. (**b**–**e**) Real-time RT-PCR validation of the four genes that showed the greatest changes in each category on the array. (**b**) *CDKN3*, (**c**) *BIRC5*, (**d**) *CDK1* and (**e**) *MKI67*. Cells were treated with 1.5 μg/ml or 3.0 μg/ml HtrA4 for 0, 24 or 48 h, n = 3. Data is expressed as mean ± SD. ^*^p < 0.05, ^**^p < 0.01, ^***^p < 0.001, ^****^p < 0.0001.

To understand how HtrA4 inhibits HUVEC proliferation, we examined the effect of HtrA4 on cell cycle gene expression using a RT2 Profiler PCR array. This array contained 84 genes which were broadly categorised into four groups (Table 1). Total RNA isolated from cells treated with vehicle control or 3.0 μg/ml HtrA4 for 24 h was analysed on the array, and a total of 35 genes across the four categories showed more than 2-fold down-regulation by HtrA4 (Table 1, Fig. 2a).

**Table 1:** Gene list of cell cycle PCR array.

Gene category	Gene name	Full name	Fold change relative to the control
G1 Phase & S Phase	*CDKN3*	Cyclin Dependent Kinase Inhibitor 3	−4.3
	*MCM4*	Minichromosome Maintenance Complex Component 4	−4.3
	*CDC25A*	Cell Division Cycle 25A	−3.8
	*SKP2*	S-Phase Kinase Associated Protein 2	−3.1
	*CDC6*	Cell Division Cycle 6	−2.6
	*MCM3*	Minichromosome Maintenance Complex Component 3	−2.6
	*MCM2*	Minichromosome Maintenance Complex Component 2	−2.4
	*MCM5*	Minichromosome Maintenance Complex Component 5	−2.4
	*CCNE1*	Cyclin E1	−2.0
	*CDK4*	Cyclin Dependent Kinase 4	−1.3
	*CCND1*	Cyclin D1	−1.2
	*CDKN1B*	Cyclin Dependent Kinase Inhibitor 1B	−1.1
	*CDK6*	Cyclin Dependent Kinase6	+1.3
G2 Phase & M Phase	*BIRC5*	Baculoviral IAP Repeat Containing 5	−5.7
	*GTSE1*	G2 And S-Phase Expressed 1	−4.2
	*CCNB1*	Cyclin B1	−3.9
	*CCNA2*	Cyclin A2	−3.6
	*STMN1*	Stathmin 1	−3.6
	*AURKB*	Aurora Kinase B	−3.5
	*RAD51*	RAD51 Recombinase	−3.0
	*CKS2*	CDC28 Protein Kinase Regulatory Subunit 2	−2.8
	*KPNA2*	Karyopherin Subunit Alpha 2	−2.8
	*MRE11A*	MRE11 Homolog, Double Strand Break Repair Nuclease	−2.2
	*CCNG1*	Cyclin G1	−1.6
	*CDK5RAP1*	CDK5 Regulatory Subunit Associated Protein 1	−1.5
	*ANAPC2*	Anaphase Promoting Complex Subunit 2	−1.3
	*SERTAD1*	SERTA Domain Containing 1	−1.1
	*MNAT1*	MNAT CDK-Activating Kinase Assembly Factor 1	−1.0
	*CCNH*	Cyclin H	−1.0
	*CDC16*	Cell Division Cycle 16	+1.2
	*CDK7*	Cyclin Dependent Kinase 7	+1.4
Cell Cycle Checkpoint & Cell Cycle Arrest	*CDK1*	Cyclin Dependent Kinase 1	−4.4
	*MAD2L1*	Mitotic Arrest Deficient 2 Like 1	−3.1
	*CDC25C*	Cell Division Cycle 25C	−3.0
	*BRCA2*	BRCA2, DNA Repair Associated	−2.8
	*WEE1*	WEE1 G2 Checkpoint Kinase	−2.8
	*BRCA1*	BRCA1, DNA Repair Associated	−2.7
	*KNTC1*	Kinetochore Associated 1	−2.5
	*CHEK2*	Checkpoint Kinase 2	−2.0
	*CHEK1*	Checkpoint Kinase 1	−1.7
	*RAD9A*	RAD9 Checkpoint Clamp Component A	−1.7
	*RAD1*	RAD1 Checkpoint DNA Exonuclease	−1.5
	*HUS1*	HUS1 Checkpoint Clamp Component	−1.5
	*MAD2L2*	Mitotic Arrest Deficient 2 Like 2	−1.3
	*RBBP8*	RB Binding Protein 8, Endonuclease	−1.3
	*ATR*	ATR Serine/Threonine Kinase	−1.3
	*CUL1*	Cullin 1	−1.2
	*CUL2*	Cullin 2	−1.2
	*CUL3*	Cullin 3	−1.1
	*CDC34*	Cell Division Cycle 34	−1.1
	*NBN*	Nibrin	−1.1
	*CCNG2*	Cyclin G2	−1.1
	*ATM*	ATM Serine/Threonine Kinase	−1.1
	*MDM2*	MDM2 Proto-Oncogene	−1.1
	*RAD17*	RAD17 Checkpoint Clamp Loader Component	+1.1
	*CDKN2A*	Cyclin Dependent Kinase Inhibitor 2A	+1.2
	*GADD45A*	Frowth Arrest And DNA Damage Inducible Alpha	+1.8
Regulation of the Cell Cycle	*MKI67*	Marker Of Prolifereation Ki-67	−4.0
	*E2F1*	E2F Transcription Factor 1	−3.7
	*AURKA*	Aurora Kinase A	−3.5
	*CCNF*	Cyclin F	−3.2
	*CCNB2*	Cyclin B2	−3.0
	*CDC20*	Cell Division Cycle 20	−2.9
	*RBL1*	RB Transcriptional Corepressor Like 1	−2.3
	*CKS1B*	CDC28 Protein Kinase Regulatory Subunit 1B	−2.2
	*CDK2*	Cyclin Dependent Kinase 2	−2.1
	*TFDP1*	Transcription Factor Dp-1	−2.1
	*CASP3*	Caspase 3	−2.0
	*CCND3*	Cyclin D3	−1.8
	*BCCIP*	BRCA2 And CDKN1A Interacting Protein	−1.6
	*BCL2*	BCL2, Apoptosis Regulator	−1.5
	*CCNC*	Cyclin C	−1.4
	*E2F4*	E2F Transcription Factor 4	−1.3
	*RB1*	RB Transcriptional Corepressor 1	−1.1
	*RBL2*	RB Transcriptional Corepressor Like 2	−1.1
	*TFDP2*	Transcription Factor Dp-2	−1.1
	*CCNT1*	Cyclin T1	−1.1
	*CDK8*	Cyclin Dependent Kinase 8	−1.0
	*CDK5R1*	Cyclin Dependent Kinase 5 Regulatory Subunit 1	−1.0
	*TP53*	Tumor Protein P53	+1.0
	*ABL1*	ABL Proto-Oncogene 1, Non-Receptor Tyrosine Kinase	+1.1
	*CDKN1A*	Cyclin Dependent Kinase Inhibitor 1A	+1.1
	*CCND2*	Cyclin D2	+1.2
	*CDKN2B*	Cyclin Dependent Kinase Inhibitor 2B	+1.2

These 35 genes were next validated by real-time RT-PCR for time and HtrA4 dose dependency using RNA samples isolated from cells treated with vehicle control, 1.5 μg/ml or 3.0 μg/ml HtrA4 for 0, 24 or 48 h. Validation of the four most highly regulated genes of each category are presented in Fig. 2b–e. *CDKN3*, which is involved in G1 and S phase of the cell cycle, was reduced 4-fold by HtrA4 on the array (Fig. 2a). RT-PCR confirmed that *CDKN3* was significantly down-regulated by HtrA4 dose-dependently at both 24 and 48 h (Fig. 2b). *BIRC5*, which is involved in the G2 and M phase of the cell cycle, showed the biggest reduction by HtrA4 on the array (Fig. 2a). RT-PCR also confirmed that *BIRC5* was significantly down-regulated by HtrA4 both dose-dependently and time-dependently (Fig. 2c). *CDK1*, which is involved in cell cycle checkpoint, was reduced 4-fold by HtrA4 on the array (Fig. 2a). This gene was also validated by RT-PCR, it was significantly down-regulated by HtrA4 in a dose- and time-dependent manner (Fig. 2d). *MKI67*, which encodes Ki67 protein that is essential for cell proliferation and cell cycle regulation, was likewise reduced by HtrA4 on the array (Fig. 2a). *MKI67* expression was again confirmed by RT-PCR to be significantly down-regulated by HtrA4 dose- and time-dependently (Fig. 2e). This data is consistent with the immunofluorescent staining of Ki67 protein presented in Fig. 1c. All these four genes showed most profound reductions in expression when cells were treated with 3.0 µg/ml HtrA4, consistent with cell proliferation data presented in Fig. 1a. All the other remaining 31 genes that were down-regulated by HtrA4 on the array (Fig. 2a), were also validated by real-time RT-PCR and the results are presented in Supplementary Fig. 1A–D.

### Isolation and validation of primary EPCs from human cord blood

**Figure 3:**
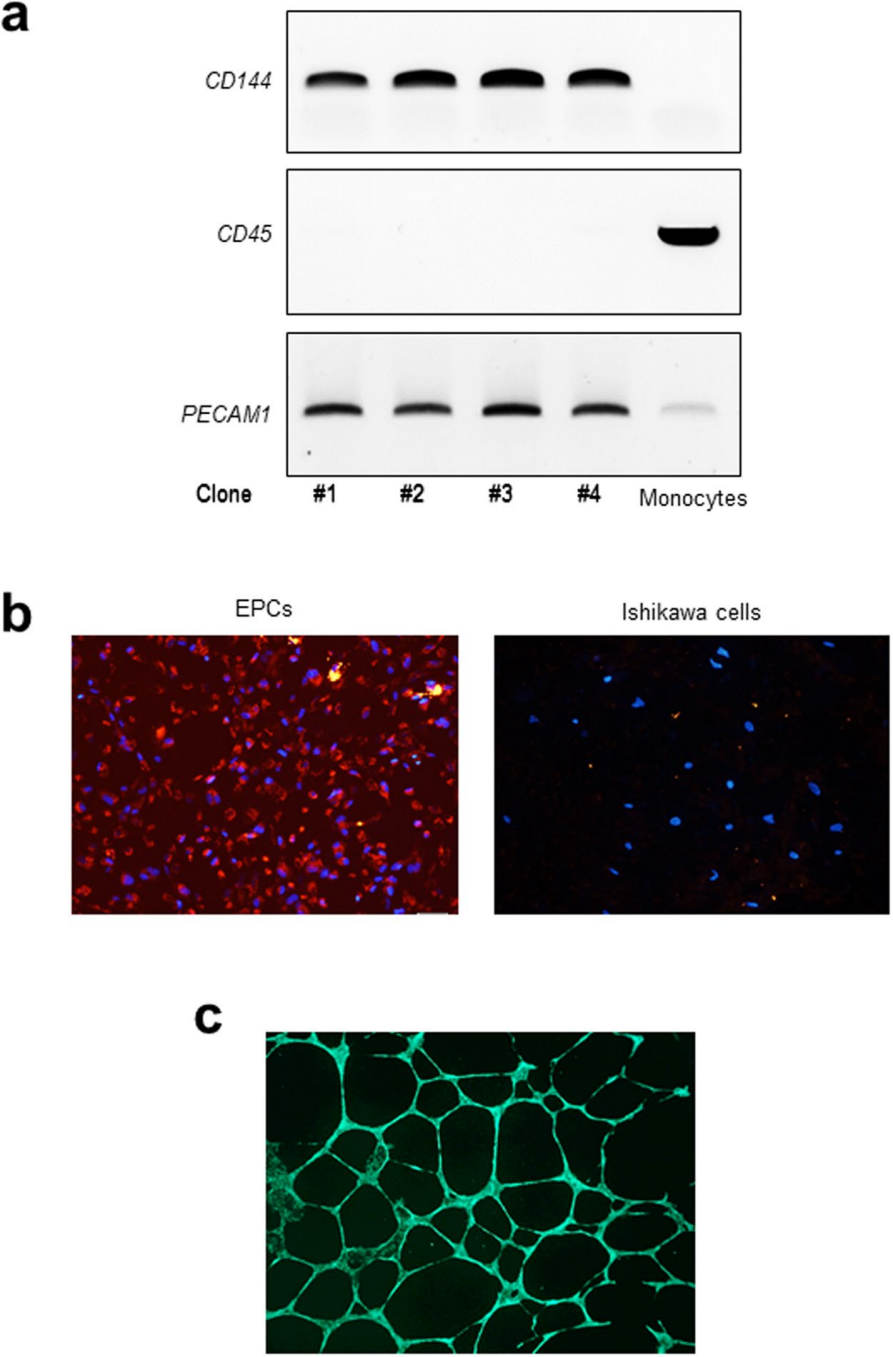
Validation of EPC clones. (**a**) RT-PCR confirmation of gene markers. CD144 and PECAM1 are positive markers of EPC, whereas CD45 is a negative marker of EPCs but a positive marker of monocytes. Full length gels are presented in Supplementary Fig. 2. (**b**) Immunofluorescent staining of Dil-labelled AcLDL (red) in EPCs. Human Ishikawa cells were used as a non-EPC control line, which did not uptake AcLDL. Nuclei were stained with DAPI (blue). Representative images at 10x magnification are shown. (**c**) Confirmation that EPCs form endothelial tubes. All EPCs formed tube-like structures on Matrigel. A representative image at 4x magnification is shown.

We next investigated the impact of HtrA4 on primary EPCs. Accordingly, EPC clones were isolated from umbilical blood from four individual pregnancies. Their identity as EPCs was verified through a combination of transcriptional and functional profiles (Fig. 3). First, gene expression of key markers was examined by RT-PCR along with a monocyte sample as a negative control (Fig. 3a). The genes examined included CD144, CD45 and PECAM1. CD144 is an EPC-specific marker, it was exclusively expressed by all four EPC clones but not by monocytes. CD45 is a monocyte-specific marker, it was negative in all four EPCs but positive in monocytes. PECAM1 is a marker for both EPCs and monocytes, it was detected in all EPC clones and monocytes (Fig. 3a).

We next examined the ability of EPCs to ingest acetylated low density lipoprotein (AcLDL), a well-recognised phenotype of EPC. All four EPC clones ingested AcLDL in culture and showed positive red staining (Fig. 3b). Ishikawa cells, which were derived from human endometrial adenocarcinoma and available in our laboratory, were used as non-EPC control cells^47,48^. These cells did not uptake AcLDL (Fig. 3b). In addition, we confirmed that all four EPC clones formed well-structured tubes (Fig. 3c). These data confirmed that the primary EPC clones isolated here were true EPCs.

### HtrA4 inhibits EPC proliferation

**Figure 4:**
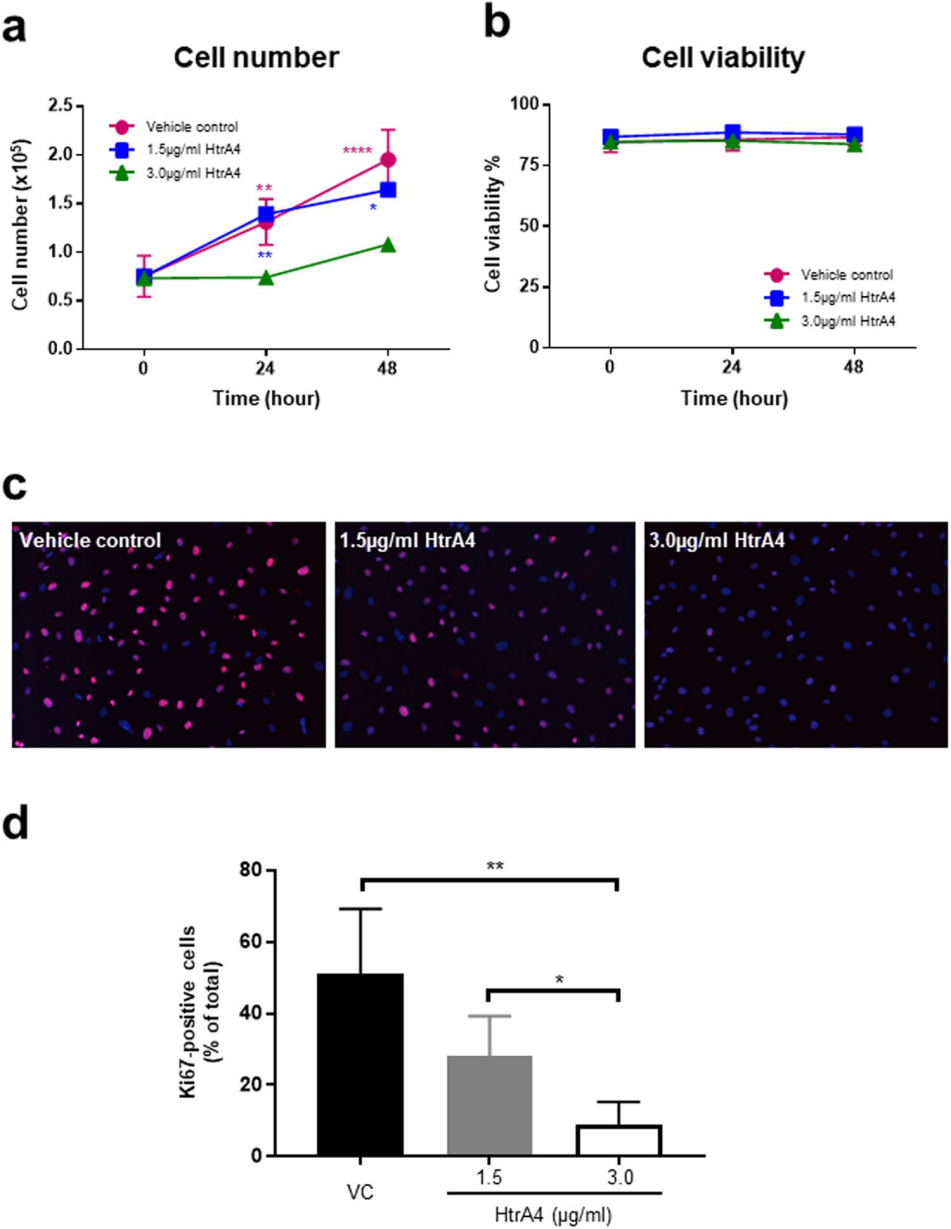
HtrA4 inhibits primary EPC proliferation. EPCs were treated with vehicle control or two doses of HtrA4 over 48 h. (**a**) Growth curve. (**b**) Percentage of viable cells. (**c**) Immunofluorescence of cell proliferation marker Ki67 at 24 h, representative images at 10x magnification are shown. (**d**) Quantification of Ki67 staining of (**c**). n = 4. Data is expressed as mean ± SD. ^*^p < 0.05; ^**^p < 0.01, ^****^p < 0.0001.

EPCs were then treated with vehicle control or two doses of HtrA4 for 0, 24 and 48 h, and cell number was counted at each time point to asses proliferation (Fig. 4a). Cells treated with vehicle control grew steadily over the 48 h period, and cell number roughly doubled at each time point as expected (Fig. 4a). Similar to HUVECs, 1.5 µg/ml HtrA4 had no obvious effect on EPC growth, whereas 3.0 µg/ml HtrA4 significantly inhibited EPC proliferation at both time points (Fig. 4a). HtrA4 again did not affect EPC viability (Fig. 4b).

The expression of proliferation marker, Ki67, was examined by immunocytochemistry (Fig. 4c). The vast majority of EPCs in the vehicle control showed clear positive nuclear staining for Ki67 (Fig. 4c), indicating that cells were in the proliferative state, consistent with our findings in HUVECs. However, the number of Ki67-positive cells was greatly reduced by HtrA4 (Fig. 4c). Quantification from four independent EPC clones confirmed that HtrA4 significantly and dose-dependently reduced Ki67 staining in EPCs (Fig. 4d), and this reduction was apparent even when HtrA4 was at 1.5 µg/ml (Fig. 4d).

### HtrA4 down-regulates cell cycle genes in EPCs

**Figure 5:**
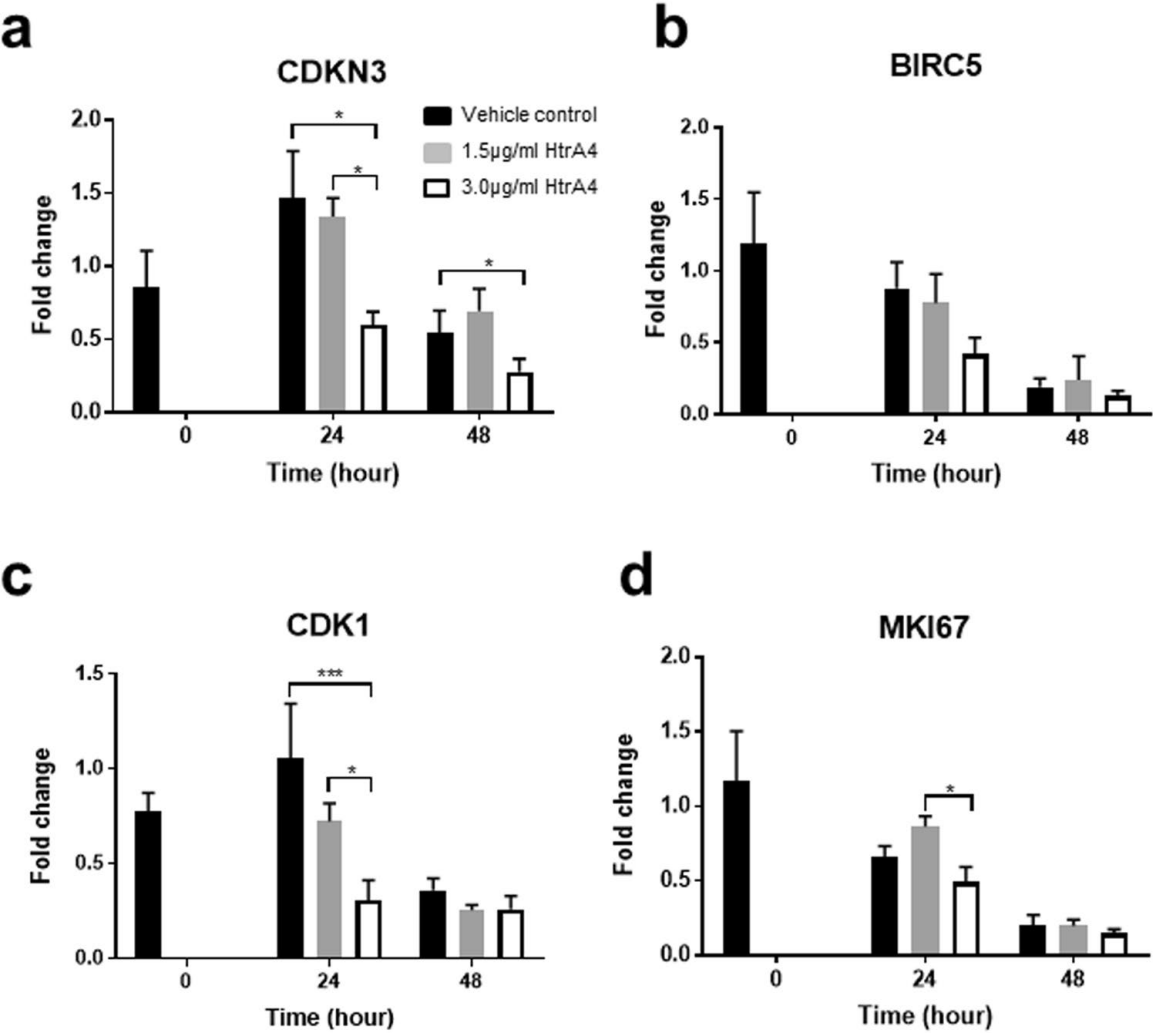
Real-time RT-PCR analysis of cell cycle genes in EPCs. (**a**) *CDKN3*, (**b**) *BIRC5*, (**c**) *CDK1* and (**d**) *MKI67*. Cells were treated with 1.5 μg/ml or 3.0 μg/ml HtrA4 for 0, 24 or 48 h, n = 4. Data is expressed as mean ± SD. ^*^p < 0.05, ^***^p < 0.001.

We next investigated whether HtrA4 also down-regulates cell cycle genes in EPCs. For this, EPCs were treated with either vehicle control or two doses of HtrA4 for 0, 24 and 48 h, and the four cell cycle genes that were highly regulated by HtrA4 in HUVECs were analysed by real-time RT-PCR (Fig. 5). *CDKN3* expression was significantly suppressed by 3.0 µg/ml HtrA4 at both 24 and 48 h (Fig. 5a). *BIRC5* expression showed a downward trend with 3.0 µg/ml HtrA4 at 24 h time point, but the difference between the treatments was not statistically significant (Fig. 5b). *CDK1* expression was dose-dependently reduced by HtrA4 at 24 h, and the greatest reduction was detected in cells treated with 3.0 µg/ml HtrA4 (Fig. 5c). Cells treated for 48 h had much lower *CDK1* expression in all treatment groups (Fig. 5c). *MKI67* expression was lowest in EPCs treated with 3.0 µg/ml HtrA4 at 24 h (Fig. 5d), and it was greatly reduced across all treatment groups at 48 h (Fig. 5d).

### HtrA4 prevents EPCs from forming endothelial tubes

**Figure 6:**
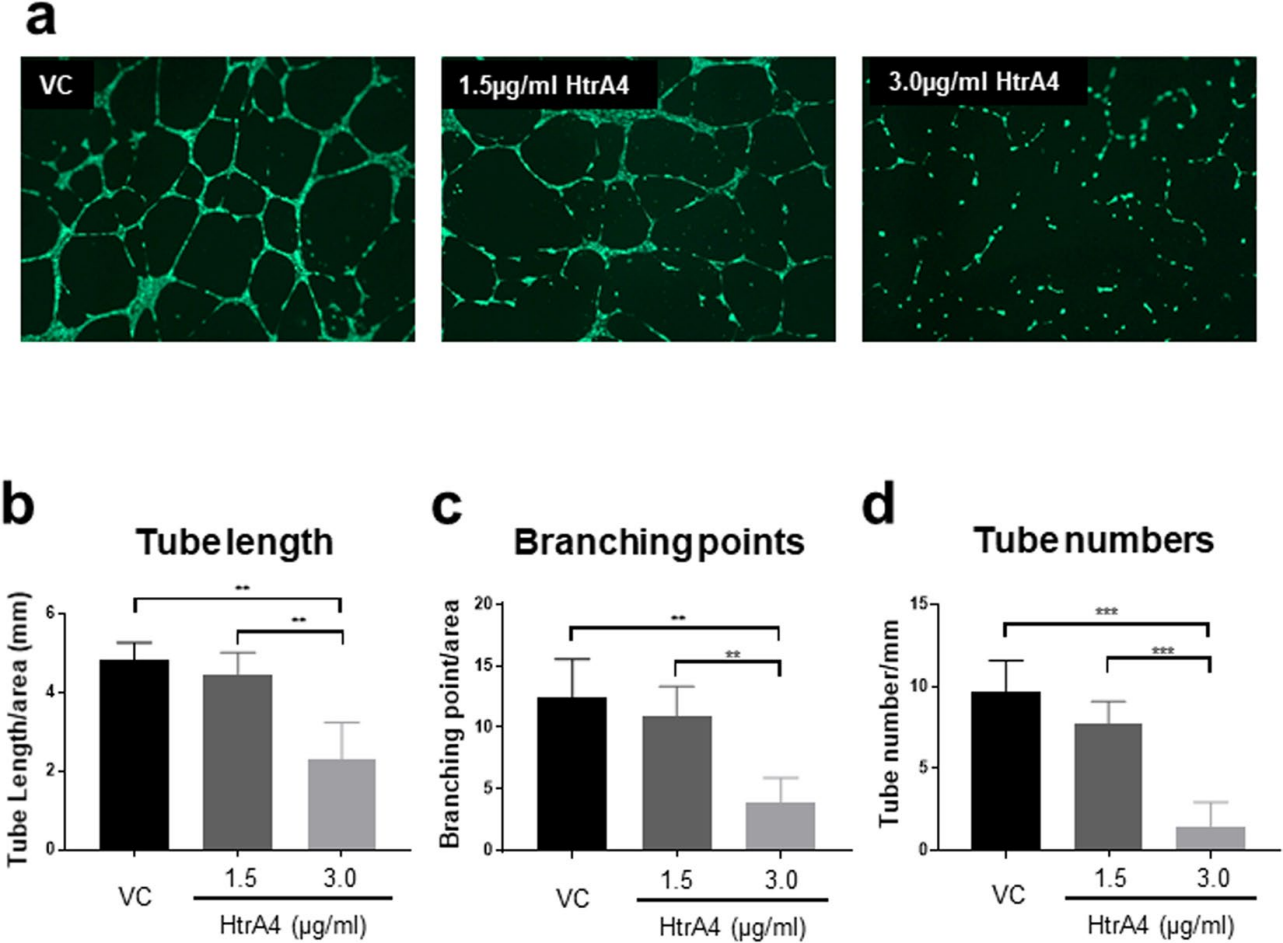
HtrA4 disrupts tube formation in EPCs. Cells were treated with vehicle control or two concentrations of HtrA4, and their abilities to form tubes were assessed. Four independent EPC clones were examined. (**a**) Representative images at 4x magnification. (**b–d**) Quantification of tube formation. (**b**) Total tube length per mm^2^ area, (**c**) Total branching point per mm^2^ area, and (**d**) Number of tubules per mm^2^ area. n = 4 for all. Data are expressed as mean ± SD. ^**^p < 0.01, ^***^p < 0.001.

We further investigated whether HtrA4 would disrupt EPC functionality using the tube formation assay. EPCs treated with vehicle control formed regular tube-like structures (Fig. 6a). EPCs treated with 1.5 µg/ml HtrA4 also formed tubes, but they were thinner and often disjointed compared to the vehicle control (Fig. 6a). In contrast, cells treated with 3.0 µg/ml HtrA4 failed to form any tubes (Fig. 6a). To quantify this result, we measured total tube length (Fig. 6b), branching point (Fig. 6c) and total number of tubes (Fig. 6d) from all four EPCs. All three parameters showed that 3.0 µg/ml HtrA4 significantly suppressed EPCs from forming endothelial tubes.

## Discussion

Early-onset PE is associated with wide-spread endothelial injury and dysfunction. Emerging evidence suggests that endothelial repair mechanisms are also compromised in early-onset PE. However, the underlying mechanisms are not well understood. HtrA4 is a serine protease that is specifically expressed by the placenta and significantly up-regulated in early-onset PE^36,40–44^. Data presented in this study suggest that high levels of HtrA4 in the circulation may impede endothelial repair. We demonstrated that high levels of HtrA4 inhibited HUVEC proliferation and significantly suppressed a number of genes that are critical for cell cycle. We also showed that HtrA4 inhibited the proliferation and differentiation of freshly isolated primary EPCs.

We first demonstrated that 3.0 µg/ml HtrA4, which represents the highest blood concentration detected in early-onset PE, significantly inhibited HUVEC proliferation. On the other hand, cells treated with either vehicle control or the lower concentration of HtrA4 grew normally. As cell viability was not affected, the high concentration of HtrA4 reduced cell growth not because of cell death but due to inhibition of cell proliferation. This was confirmed by immunostaining for proliferation marker Ki67. Very little Ki67 staining was detected in cells treated with 3.0 µg/ml HtrA4 compared to the vehicle control. Although not statistically significant, cells treated with 1.5 µg/ml HtrA4 also showed less Ki67 staining, suggesting that even lower concentrations of HtrA4 may restrain endothelial cell proliferation.

We further demonstrated that HtrA4 dose-dependently down-regulated a number of cell cycle genes by a PCR array and by real-time RT-PCR. As the original PCR array experiment did not include replicates, statistical testing could not be performed. This limitation was, however, mitigated by extensive validation of the findings by real-time RT-PCR on three independent samples per treatment. Among 84 cell cycle genes examined on the array, HtrA4 down-regulated 35 genes by more than 2-fold, these changes were subsequently confirmed by RT-PCR. Overall, HtrA4 dose-dependently down-regulated genes that are critical for cell cycle, and 3.0 µg/ml HtrA4 had the most profound effect on their expression. *MKI67* mRNA expression was significantly reduced by HtrA4 even at the lower dosage at both 24 and 48 h, supporting the immunocytochemistry result. The data presented in this study suggests that at the molecular level, HtrA4 dose-dependently inhibited the expression of endothelial genes that are involved in proliferation. Among the 49 genes that showed less than 2-fold changes on the array and were thus not further examined by RT-PCR, 3 showed an upward (fold change >1.3) whereas 7 showed a downward (fold change >1.5) trend following treatment with HtrA4 (Table 1). These borderline genes may warrant further investigation in the future. Two apoptosis-related genes, *BCL2* and *CASP3*, were not altered by HtrA4 treatment, consistent with the cell viability data.

Our data presented in this study also demonstrated that HtrA4 likewise inhibited EPC proliferation and expression of genes involved in cell cycle. Firstly, we isolated four EPC clones from four individual pregnancies and confirmed their identity. This was achieved by determining the mRNA expression of EPC markers, their ability to uptake AcLDL, and to form endothelial tubes on Matrigel. We then used these primary EPCs and demonstrated that high concentrations of HtrA4 inhibited EPC growth, consistent with the HUVEC result. Similarly, HtrA4 did not affect EPC viability, again indicating that HtrA4 did not induce cell death. Immunostaining for Ki67 showed that HtrA4 reduced the number of Ki67-positive cells in a dose-dependent manner in all four EPCs. Compared to the HUVECs, EPCs appeared to be more sensitive to HtrA4, because a statistically significant reduction in Ki67 staining was observed in EPCs but not in HUVECs when they were treated with 1.5 µg/ml HtrA4. Furthermore, the effect of 3.0 µg/ml HtrA4 on Ki67 staining was more pronounced in EPCs than in HUVECs. This result suggests that HtrA4 even at lower dosages may restrain EPC numbers and function in normal pregnancy; its significance is unclear but it may be a mechanism that permits maternal vascular remodelling/adaptation for pregnancy.

The four cell cycle genes, *CDKN3*, *BIRC5*, *CDK1* and *MKI67* that were highly down-regulated by HtrA4 in HUVECs, were also examined in EPCs. All these genes except *BIRC5* showed a significant reduction in expression when EPCs were treated with 3.0 µg/ml HtrA4 for 24 h. At 48 h, only *CDKN3* expression was significantly down-regulated by 3.0 µg/ml HtrA4. However, at the 48 h time point, expression of all four genes was significantly lower even in the vehicle control. This data suggests that EPCs proliferate more quickly than HUVECs, and they may have stopped proliferating by 48 h. Therefore, the impact of HtrA4 treatment on expression of *BIRC5*, *CDK1* and *MKI67* was less obvious at 48 h. In EPCs, while Ki67 immunostaining was reduced by HtrA4 in a dose-dependent manner, the lower dosage of HtrA4 had no significant effect on *MKI67* gene expression, suggesting that HtrA4 may also regulate Ki67 at the protein level.

One of the main functions of EPCs is to differentiate into resident endothelial cells to repair injured endothelium^49^. An *in vitro* test of this differentiation process is to examine their angiogenic ability to form tube-like structures on Matrigel, which we have successfully confirmed for all four primary EPC clones. However, when these EPCs were treated with 1.5 µg/ml HtrA4, tubes were formed but they were thinner and disjoined in many areas, suggesting that even the lower concentration of HtrA4 affected EPC function. When EPCs were treated with 3.0 µg/ml HtrA4, the tube formation process was completely blocked.

One limitation of our study was the use of HUVECs as a model. As HUVECs may not reflect all features of human vascular endothelial cells, future studies are warranted with endothelial cells of other vascular origin. However, we validated the HUVEC data in primary EPCs in this study. Another limitation was the source of EPCs, which ideally would be from peripheral blood, however, EPCs are rare cell populations in peripheral blood and generating EPC clones is a difficult and long process. As EPCs from either maternal and fetal origin exhibit similar phenotypes and characteristics once they are in culture^46^, in this study we used umbilical cord blood derived EPCs due to their relative abundance. Furthermore, since HtrA1 is also significantly elevated in early-onset PE circulation^50^, whether HtrtA1 has a similar function or work synergistically with HtrA4 should be investigated.

In summary, this is the first study to show that placental-derived HtrA4, which is elevated in the early-onset PE circulation may inhibit endothelial cell proliferation. Furthermore, our study demonstrated that high levels of HtrA4 may also impede endothelial repair by inhibiting the proliferation and differentiation of circulating EPCs. These results suggest that circulating HtrA4 may present a potential therapeutic target for treatment of early-onset PE.

## Material and Methods

### Cell culture

HUVECs (ATCC, Maryland, USA) were cultured at 37 °C in a humidified atmosphere of 5% CO_2_ in air, and maintained in DMEM (Thermo Fisher Scientific, VIC, Australia) supplemented with 1% antibiotics (Thermo Fisher Scientific), 2 mM L-glutamine (Sigma-Aldrich, Missouri, USA), 1 mM sodium pyruvate (Thermo Fisher Scientific) and 10% fetal bovine serum (FBS, Thermo Fisher Scientific). The starting passage of the HUVECs was 12, and the experiments were completed within eight passages. Cells were sub-cultured in 12-well plates (Thermo Fisher Scientific) at a density of 1.0 × 10^5^ per well for 24 hours (h), then treated with two doses of recombinant HtrA4 (1.5 µg/ml and 3.0 µg/ml, BioTeZ, Berlin, Germany) or vehicle control for 24 or 48 h. The vehicle control contains 150 mM NaCl, 5 mM CaCl2, 50 mM Tris-HCl pH 7.5, 0.05% Brij 35 solution, 50 mM imidazole in ultrapure water. The two doses of HtrA4, 1.5 µg/ml and 3.0 µg/ml, were chosen to represent the median and highest levels of HtrA4 detected in the early-onset PE circulation^36^.

### Cell proliferation assay

HUVECs were plated in 12-well plates and treated with either vehicle control or two doses of HtrA4. Cell numbers and viability in each treatment were determined at 0, 24 and 48 h post-treatment. Cells from each well were trypsinized and re-suspended in 500 µl growth media, 10 µl of the cell suspension was mixed with an equal volume of trypan blue which stains dead cells. The total number of viable (clear cytoplasm) and dead (blue cytoplasm) cells were then counted using an automated cell counter (Countess, Thermo Fisher Scientific). Cell viability was determined by the percentage of viable cells in the total number of cells collected from each treatment. Cells were subsequently collected for RNA isolation and the experiment was repeated independently three times.

### RNA extraction

RNA was extracted using an RNeasy Mini Kit (Qiagen, Hilden, Germany) and genomic DNA was removed using an RNase-free DNase set (Qiagen) according to the manufacturer’s protocols. RNA was isolated from three independent experiments and concentration was determined using Nanodrop ND-1000 (Thermo Fisher Scientific).

### Analysis of cell cycle genes using a pathway-specific PCR array

RNA was isolated from HUVECs treated with either vehicle control or 3.0 μg/ml HtrA4 for 24 h. Samples from three independent experiments were pooled, and 500 ng of pooled RNA representing control and HtrA4 treatment was used for the array. RNA was reverse transcribed into complementary DNA (cDNA) using a RT^2^ First Strand Kit (Qiagen), and applied onto a RT^2^ Profiler 84 gene PCR array (Qiagen) according to manufacturer’s instructions on an ABI 7900 HT Fast real-time machine (Applied Biosystems, VIC, Australia). Results were then analysed using Qiagen RT^2^ profiler PCR array data analysis software (Qiagen). Genes showing more than 2-fold differences in expression between the vehicle control and 3.0 µg/ml HtrA4 treatment were validated by real-time PCR.

### Real-time RT-PCR analysis

For RT-PCR validation, cells were treated with 1.5 μg/ml or 3.0 μg/ml HtrA4 for 0, 24 and 48 h respectively, and RNA (300 ng) from each treatment of three independent experiments was reverse transcribed with a SuperScript III First-Strand kit (Thermo Fisher Scientific) according to manufacturer’s instructions. Real-time PCR was performed with primers specified in Supplementary Table 1 on an ABI 7900 HT Fast real-time machine (Applied Biosystems, California, USA) with the following conditions: 1) 95 °C for 10 min for enzyme activation, 2) 40 cycles of denaturation (15 s at 95 °C), annealing (5 s at 58 °C), extension (10 s at 72 °C), and a single fluorescence measurement at 70–75 °C for quantitation, and 3) dissociation curve assessment between 60 °C and 95 °C with continuous fluorescence measurement. All cDNA samples were diluted 1:80 and PCR reaction was prepared to a final volume of 10 µl with 5 µl SYBR Green PCR Master Mix (Thermo Fisher Scientific), 4 µl diluted cDNA sample and 0.5 µM final concentration of forward and reverse primers. All samples were run in triplicates, gene expression levels were normalised to 18S, and fold changes were calculated using ΔΔCt.

### Immunocytochemistry

Cells were seeded on 10-mm glass coverslips (Thermo Fisher Scientific) that were placed in 48-well plates, and treated with vehicle control or two doses of HtrA4 for 24 h. Cells were fixed with 4% paraformaldehyde (VWR, Radnor, USA) for 10 min and permeabilised with 0.1% Triton X-100 (Sigma-Aldrich) in PBS for 5 min. All procedures from here onwards were carried out at room temperature unless stated otherwise. Cells were blocked with 1% BSA in PBS for 2 h, incubated at 4 °C overnight with an anti-Ki67 rabbit monoclonal antibody diluted 1:250 (cat# ab16667, Abcam, Cambridge, United Kingdom), and then with a donkey anti-rabbit Alexa Fluor 568 antibody diluted 1:200 (cat# A10042, Thermo Fisher Scientific) for 2 h. Nuclei were stained with 5 µg/ml 4′,6-diamidino-2-phenylindole dihydrochloride (DAPI, Sigma-Aldrich) for 10 min, and coverslips were mounted onto glass slides with fluorescent mounting media (Dako, Glostrup, Denmark). Staining was visualised using an Olympus BX60 fluorescent microscope (Olympus, Notting Hill, Australia) and images were taken at 10x magnification using an Olympus DP70 camera and Olympus CellSens software (Olympus). Three to four images per each treatment were taken randomly. Ki67 positive cells were quantified using FIJI software (NIH, Maryland, USA). Experiments were repeated independently three times.

### Isolation of primary EPCs from umbilical cord blood of term human pregnancy

Umbilical cord blood from healthy term human pregnancy was collected at Monash Medical Centre (Melbourne, Australia) with approval from the Monash Medical Centre Human Research Ethics Committees (HREC reference number: 13357B) and all experiments were performed in accordance with the relevant guidelines and regulations. Informed consent was obtained from all study participants. Primary EPCs were isolated from the cord blood as previously described^46^. Briefly, mononuclear cells were separated from blood using Ficoll-Paque PLUS (Amersham Biosciences, Little Chalfont, United Kingdom) and centrifugation. They were then seeded at a 3–5 × 10^7^ per well density in 6-well plates that were pre-coated with rat tail collagen I (Merck, New Jersey, USA), and maintained in complete EGM-2MV BulletKit media (EGM-2, Lonza, Basel, Switzerland) containing 10% FBS and 1% antibiotics. Media were changed daily till colonies of endothelial cells were observed. Individual colony was then isolated using a cloning cylinder (Sigma-Aldrich) and propagated as monoclonal EPC line in complete EGM-2 media.

### Validation of primary EPCs

To confirm the purity and identity of EPC clones, RNA was extracted from each clone and reversed transcribed into cDNA using SuperScript III First-Strand kit. RT-PCR was then performed on a conventional block PCR machine at 58 °C for 30 cycles, together with an EPC-negative cDNA sample of monocytes which were also isolated from the cord blood at the same time. Two EPC markers (CD144 and PECAM1) and one hematopoietic cell/monocyte-specific marker (CD45) were examined using the following primers: CD144, 5′-GCACCAGTTTGGCCAATATA-3′ and 5′-GGGTTTTTGCATAATAAGCAGG-3′; PECAM1, 5′-CACACCAAGAACTCTCCCCA-3′ and 5′-CCCTCACCTGTCCTGCTCAT-3′; CD45, 5′-CTGGGGAGAAGGAAAGCAAA-3′ and 5′-GCAGTGAATGAGTAGAGGTG-3′. Phenotypic characteristics of EPCs were also examined by ingestion of AcLDL. EPCs were seeded in 48-well plates at a density of 0.5 × 10^5^ per well for 24 h, and then incubated with 10 µg/ml Dil-complexed AcLDL (Thermo Fisher Scientific) for 4 h at 37 °C. The cells were counterstained with DAPI and visualised using a fluorescent microscope. Cells that ingested AcLDL fluoresced red. Another major functional characteristic of EPCs is forming tube-like structures on Matrigel, to confirm this, EPCs were cultured for 24 h at a density of 1.0 × 10^5^ per well in 48-well plates that were pre-coated with growth factor reduced Matrigel (BD Bioscience, New Jersey, USA), and stained with 4 µg/ml calcein AM fluorescent dye (BD Bioscience) for 30 min at 37 °C. Tube formation was visualised using a fluorescent microscope. Four clones were independently verified by all of the above methods.

### Treatment of EPCs with HtrA4

For each EPC clone, cells were sub-cultured in 12-well plates at a density of 0.5 × 10^5^ per well for 24 h, then treated with recombinant HtrA4 (1.5 μg/ml or 3.0 μg/ml) or vehicle control for 24 or 48 h. At the end of the treatment, cell number was measured as described per HUVEC proliferation assay protocol, and cell viability was determined by calculating the percentage of viable cells over the total number of cells collected per treatment. RNA was subsequently extracted as described per HUVEC experiment and real-Time RT-PCR was performed using selected primers specified in Supplementary Table 1. EPCs were also seeded at a density of 0.5 × 10^5^ per well on 10-mm glass coverslips that were placed in 48-well plates, treated with vehicle control or two concentrations of HtrA4 for 24 h, and then stained with a Ki67 antibody as per HUVEC experiment. All experiments were repeated with four individual EPC clones.

### Assessment of EPC tube formation following HtrA4 treatment

EPCs were seeded at a density of 1.0 × 10^5^ per well in 48-well plates that were pre-coated with growth factor reduced Matrigel, and treated with either vehicle control or two doses of HtrA4 (1.5 µg/ml or 3.0 µg/ml) for 16 h. Cells were washed three times with Hanks’ balanced salt solution (Thermo Fisher Scientific) and labelled with 4 µg/ml calcein AM fluorescent dye for 30 min at 37 °C. Tubes were assessed using an Olympus BX60 fluorescent microscope at 4x magnification, and images were taken using an Olympus DP70 camera and Olympus CellSens software. Three images per treatment condition were taken randomly, and the total tube length, branching points and total tube numbers per area were quantified using FIJI software. Experiments were repeated with four individual EPC clones.

### Statistical analysis

Statistical analyses were conducted using GraphPad Prism (v.6, GraphPad Software Inc., CA). Data were expressed as mean ± SD, and compared using one-way ANOVA followed by Tukey’s post-hoc test, p < 0.05 was considered significant.

## Supplementary information

Supplementary figure 1&2, supplementary table 1

## Data Availability

All data generated or analysed during this study are included in this published article (and its Supplementary information files).
