# Supplementary material for: HtrA4 may play a major role in inhibiting endothelial repair in pregnancy complication preeclampsia
Source: Sci Rep. 2019 Feb 25;9:2728. doi: 10.1038/s41598-019-39565-9 (PMC6389976; doi:10.1038/s41598-019-39565-9)
Supplement: Supplementary file 1 — Supplementary figure 1&2, supplementary table 1 [file 41598_2019_39565_MOESM1_ESM.pdf]

HtrA4 may play a major role in inhibiting endothelial repair in pregnancy complication preeclampsia

Yao Wang[1,2], Rebecca Lim[3,4] and Guiying Nie[1,2,5*]

[1]Implantation and Placental Development Laboratory, Centre for Reproductive Health, Hudson Institute of Medical Research, Clayton, Victoria, 3168, Australia

[2]Department of Molecular and Translational Science, Monash University, Clayton, Victoria, 3800, Australia

[3]Department of Obstetrics and Gynaecology, Monash University, Clayton, Victoria, 3168, Australia

[4]Australian Regenerative Medicine Institute, Monash University, Clayton, Victoria, 3168, Australia

[5]Department of Biochemistry and Molecular Biology, Monash University, Clayton, Victoria, 3800, Australia

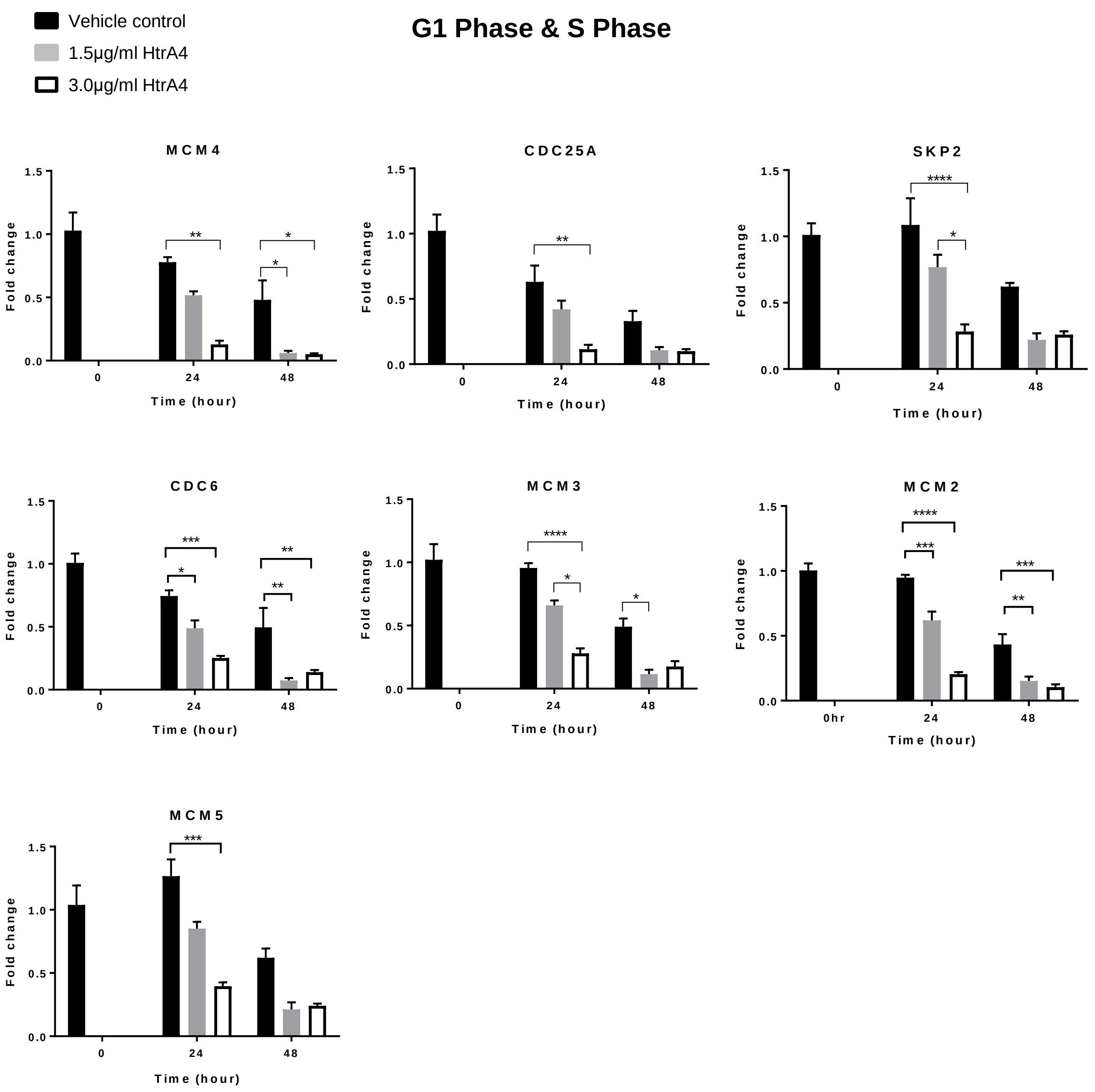

Supplementary Figure 1A

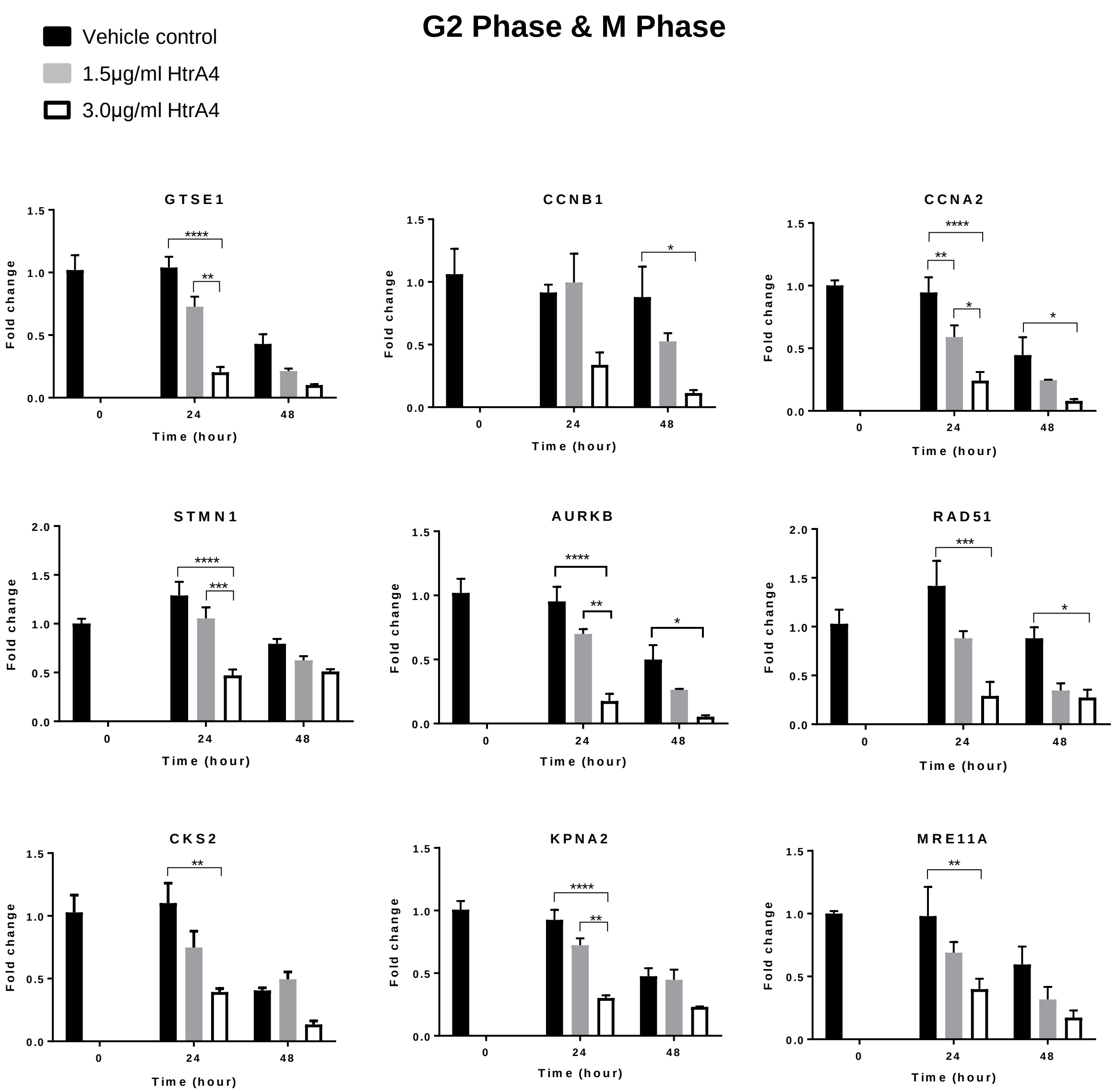

Supplementary Figure 1B

Cell Cycle Checkpoint and Cell Cycle Arrest

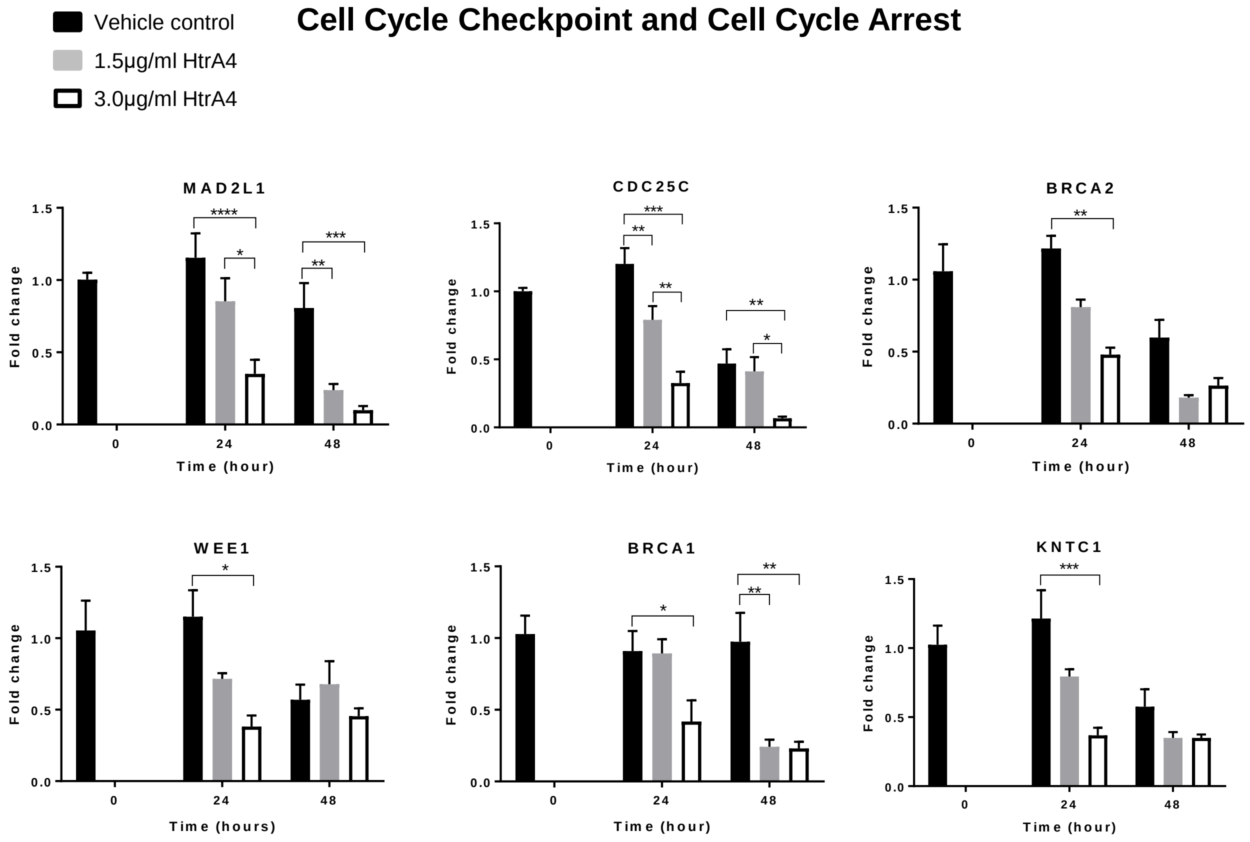

Supplementary Figure 1C

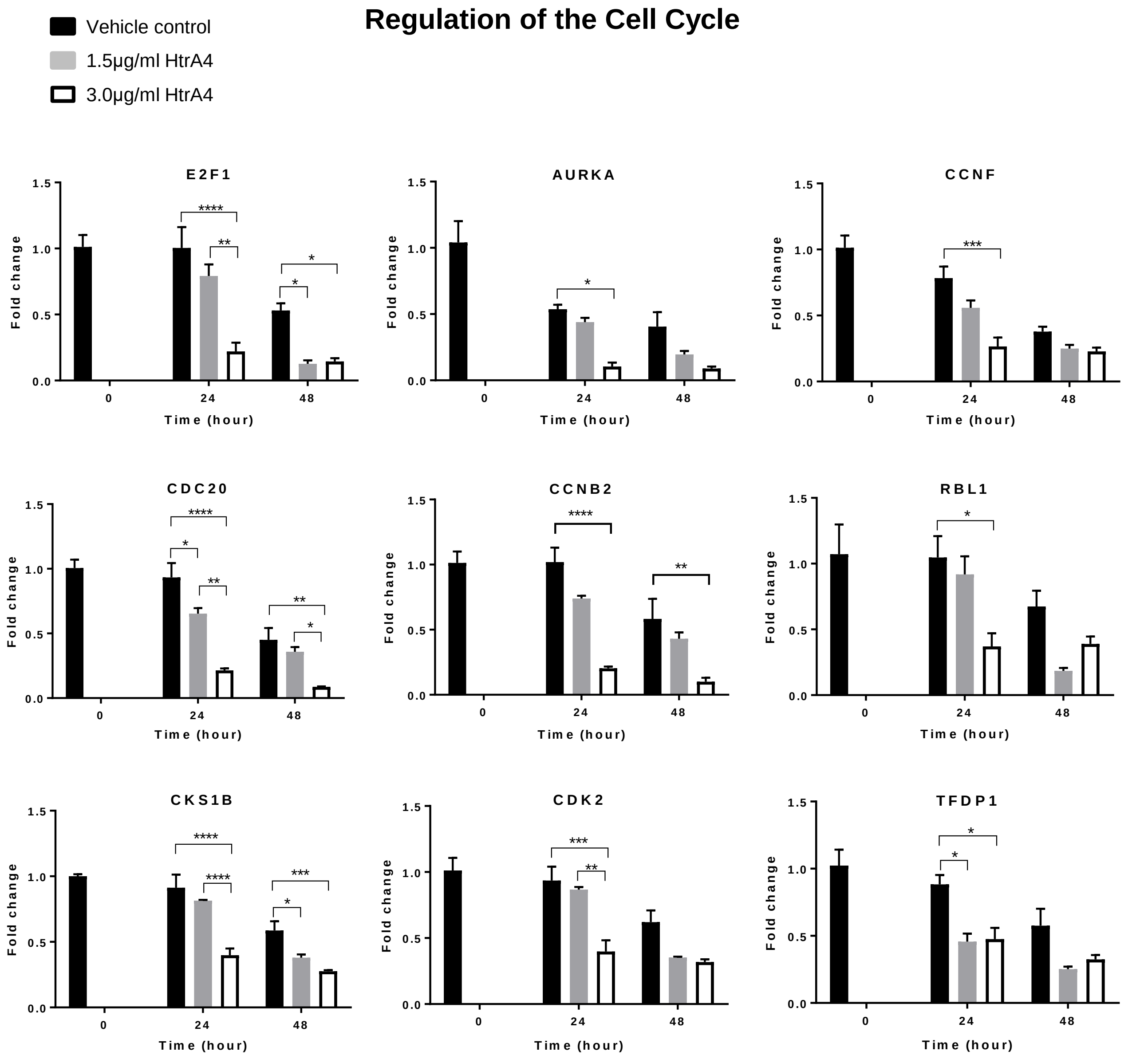

Supplementary Figure 1. Real-time RT-PCR validations of cell cycle genes represented in grey bar in Figure 2A. A) Genes involved in G1 and S phase. (B) Genes involved in G2 and M phase. (C) Genes involved in cell cycle checkpoint and cell cycle arrest. (D) Genes involved in regulation of the cell cycle. Cells were treated with 1.5μg/ml or 3.0μg/ml HtrA4 for 0, 24 or 48h, n=3. Data is expressed as mean ± SD. *p<0.05, **p<0.01, ***p<0.001, ****p<0.0001.

Supplementary Figure 1D

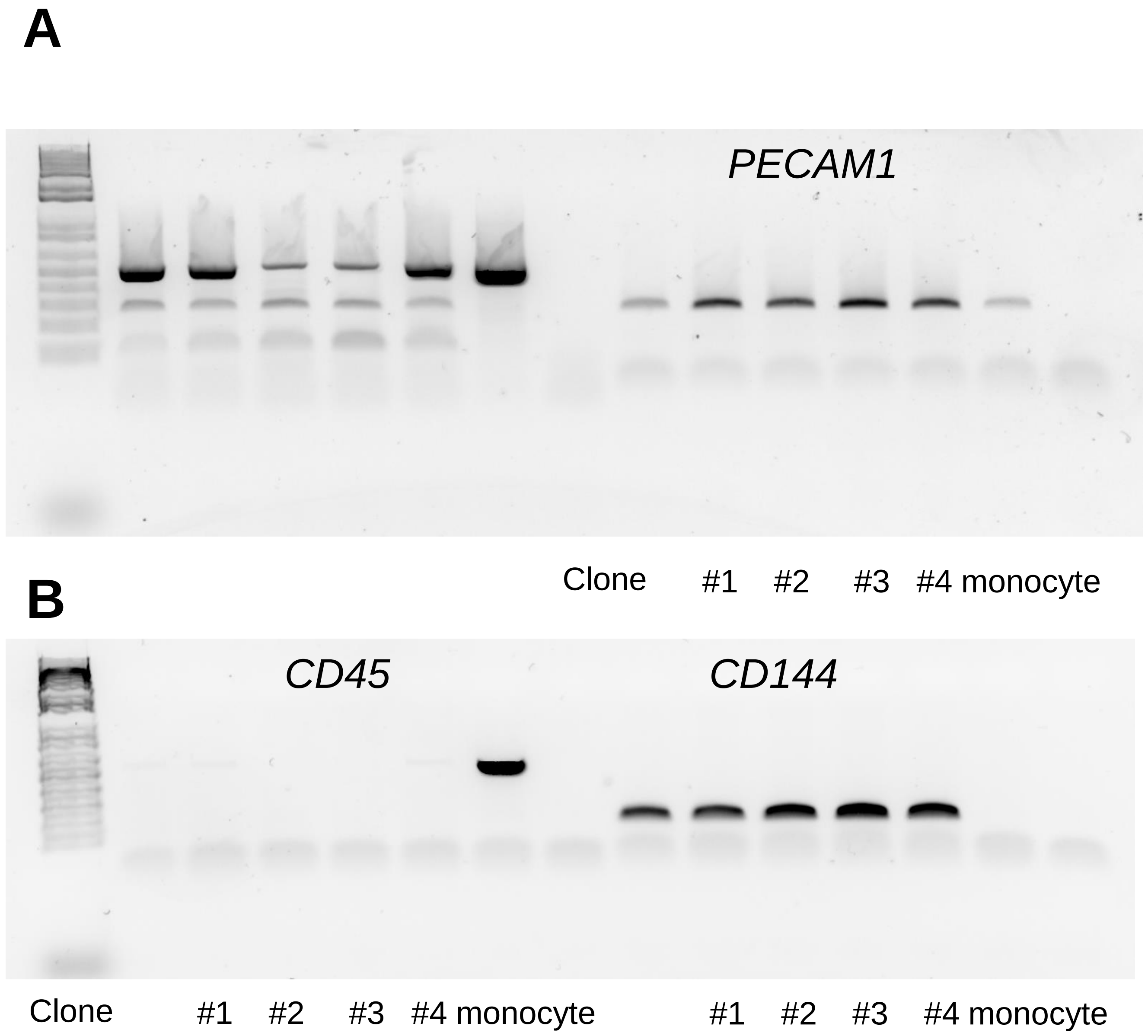

Supplementary Figure 2. Full PCR agarose gel electrophoresis images of Figure 3A. (A) Full image of PECAM1 on the right, including negative sample in last lane. (B) Full image of CD45 and CD144. Also included negative sample in last lane for each primer.

Supplementary Table 1

Gene symbol	Accession Number	Primer sequence (5'→3')	Product Size (bp)
AURKA	NM_198433.1	Forward TGACCCCGATCAGTTAAGGA<br>Reverse CGGACAGACACACACAGCATTC	197
AURKB	NM_004217	Forward TGGACCTAAAGTTCCCCGCT<br>Reverse ACGCACCCGAGTGAATGACA	200
BIRC5	NM_001168.2	Forward CCATCCTTAAAACCAGACCC<br>Reverse TAACCTGCCATTGGAACCTC	218
BRCA1	NM_007294	Forward GCTGTTGCTTTCTTTGAGGT<br>Reverse CAATCAAGTCTTCACTGCCC	196
BRCA2	NM_000059	Forward ATGGAATGAGGTCTCTTAGT<br>Reverse TCAAAAGGAAACACCACTCT	192
CCNA2	NM_001237.3	Forward TGGTGGTCTGTGTTCTGTGA<br>Reverse CCATCTGTTCTGTGATTTTTT	211
CCNB1	NM_031966	Forward TCCTTTTGGTTTACCTGGGG<br>Reverse TAGGGATTCGGTGGTAGACT	216
CCNB2	NM_004701	Forward GCCAAGAATGTGGTGAAAGT<br>Reverse AGAAAAAGGGCACAATGAAG	202
CCNF	NM_001761	Forward CCCCCAAATGCTCTTGAACC<br>Reverse CGACAGGACGGGTCTACAGT	196
CDC6	NM_001254	Forward GAACTCCTGACCCTCAAGTG<br>Reverse CCAGTGTCCTCCAAATCTCC	181
CDC20	NM_001255	Forward GGTGGCTGAACTCAAAGGTC<br>Reverse CTGAGGTGATGGGTTGGTCT	216
CDC25A	NM_001789	Forward CTACTCATCCCTGCCCTCTG<br>Reverse GTCCTCTCCCCCACATTTTT	217
CDC25C	NM_001790	Forward TGGTGGCAGAGTCCTGGAG<br>Reverse AAGAGGGGGAAACAAAGAAG	205
CDK1	NM_001786.4	Forward AAAGCTAACATGAGAGCAT<br>Reverse CGAAAGCCAAGATAAGCAACT	193
CDK2	NM_001798	Forward CTGAGACAGGGATTGTGCTT<br>Reverse CTATGGGGTAGGAGGTGGAC	164
CDKN3	NM_005192.3	Forward ACAGCCTGCGAGACCTAAGA<br>Reverse AACACTGGTGGTTTCATTTC	218
CKS1B	NM_001826	Forward AGTAGAGCCACCACCACCAT<br>Reverse TCCGCAAGTCACCACACATA	220
CKS2	NM_001827	Forward CTGAAGAGGAGTGGAGGAGA<br>Reverse GCACAGGTATGGATGAAAGA	235
E2F1	NM_005225	Forward GCTGTTCTTCTGCCCCATAC<br>Reverse TTCACCACCCCTCCTGCCCA	222
GTSE1	NM_016426	Forward CCTGTTTGGAGCGGGGCATC<br>Reverse TCTGGGGTTGGGTGGGGGAT	268
KNTC1	NM_014708	Forward AGGAGTTTGGGATTTTGGCA<br>Reverse CAGAATTTCACAGGGAGCAG	197
KPNA2	NM_002266	Forward GCCGTGACCAACTATACCAG<br>Reverse TGTCTAAGCCTCCACATTCT	217
MCM2	NM_004526	Forward GCGTATTCAGGCTGCTTTTG<br>Reverse GATAACTGTGGCGAGTGGAT	193
MCM3	NM_002388	Forward TGGGGGCACAACTGTTTTCT<br>Reverse CATCTCGGATATTCATCTGG	218
MCM4	NM_182746	Forward GCACCCAGCCTTTGTTTTAT<br>Reverse GCCAGGTAACCAAGTATTATTCC	196
MCM5	NM_006739	Forward ACTTCACCAAGCAGAAATAC<br>Reverse GCAGAGGTCCCAGCAACAT	205
MAD2L1	NM_002358	Forward CTGTAGATGGAAAAACTTGTGC<br>Reverse AAATGAAGGTCAAAAGGAGC	214
MKI67	NM_002417.4	Forward TTGGTACTGGGGGAGGGAGA<br>Reverse TGGGAGGCGAAAAAGTAAAA	188
MRE11A	NM_005591	Forward GCACTGATGGAATCCCTCTA<br>Reverse AACAGGCTGAACCAAATGAA	312
RAD51	NM_002875	Forward TGTAGCAAAGGGAATGGGTC<br>Reverse GCAGGTAGATGGTGAAGGGC	159
RBL1	NM_183404	Forward GGACCAGTGAAAGAGGAAAG<br>Reverse TGTGCGGGGAAATATAAATG	211
SKP2	NM_005983	Forward ACATTTCAGCCCTTTTCGTG<br>Reverse GAGAATCCAGAACACCCAGA	204
STMN1	NM_203401.1	Forward AAGACGCAAGTCCCATGAAG<br>Reverse CCATTTGTGCCTCTCGGTT	173
TFDP1	NM_007111	Forward CTAAACAGCCCCAAGCAAAC<br>Reverse CTCCATAGCCGCACCAGCAT	220
WEE1	NM_003390	Forward CCCTCCTTTGGAATGCTGTA<br>Reverse TGTGAAAAACTTGGAATGTC	251
